# Nutrigenetics in Disease Prevention: Mechanistic Pathways and Risk Biomarkers

**DOI:** 10.3390/nu17132165

**Published:** 2025-06-29

**Authors:** Ruth Birk

**Affiliations:** Nutrition Department, Health Science Faculty, Ariel University, Ariel 40700, Israel; ruthb@ariel.ac.il

The fields of nutrigenetics and nutrigenomics are rapidly changing our understanding of how individual genetic variations influence the body’s response to nutrients and dietary patterns. The related research field studies the interplay between our genes and our environment—particularly our nutrition. Growing evidence shows the effect of gene–environment and gene–gene interactions on metabolic health, disease susceptibility, and therapeutic outcomes.

Recent studies demonstrate that genetic variants such as those in *CYP2R1*, *TMEM18*, *FTO*, and Vitamin D metabolic pathways can significantly modulate the risk of obesity, metabolic syndrome, and other diet-related conditions. Importantly, these risks are not deterministic; rather, lifestyle factors such as physical activity, alcohol consumption, and dietary composition can either amplify or mitigate genetic predispositions. This paradigm reinforces the idea that personalized nutrition, informed by genetic screening, offers promise for targeted treatment and preventive strategies that are proactive and precise.

Furthermore, insights into gene–gene interactions add another layer of complexity and opportunity. Understanding how multiple variants interact to impact nutrient metabolism and disease progression opens up new avenues for research and clinical application. As we enhance our ability to map these interactions, we move closer to a healthcare model that is predictive, preventive, and personalized.

The implications for screening are significant. Genetic profiling could soon become a routine tool in nutritional assessment, allowing clinicians to identify at-risk individuals and tailor interventions accordingly. Similarly, treatment strategies may increasingly depend on nutrigenomic data to optimize dietary therapy, improve drug efficacy, and reduce adverse effects.

This evolving landscape calls for multidisciplinary collaboration among geneticists, nutritionists, clinicians, and data scientists to further unlock the full potential of nutrigenetics and nutrigenomics to transform public health—shifting the focus from disease management to health optimization through personalized nutrition.

Nutrigenetics and nutrigenomics literacy is becoming necessary for clinical nutritionists, healthcare professionals, and researchers as the role of gene–diet interactions in health and disease becomes clearer. An understanding of nutrigenetics and nutrigenomics enables professionals to interpret genetic data accurately, apply evidence-based personalized nutrition strategies, and contribute to advancing research in precision nutrition.

The studies in this section, summarized below, represent cutting-edge examples of how gene–diet interactions are being translated into practical health insights.

FGF21 genetics, protein intake, and NAFLD risk:

Lee et al. investigated how genetic variants in the FGF21 pathway affect the risk of non-alcoholic fatty liver disease (NAFLD) and how these effects are modified by dietary protein intake. Using a polygenic hazard score, they found that individuals (particularly females) with lower genetic risk interact with dietary protein intake to elevate NAFLD risk, emphasizing the need for genotype-guided dietary interventions.

FTO common obesity SNPs interact with actionable environmental factors:

Chermon & Birk analyzed, in a population-based study, the interaction between common obesity-associated FTO gene polymorphisms and lifestyle factors such as physical activity, sugar-sweetened beverage (SSB) consumption, and wine intake. Adult carriers of the rs9939609 risk allele had higher obesity risk, but this risk was significantly mitigated by physical activity and moderate wine consumption. Conversely, SSB intake exacerbated the genetic risk. These findings underscore the importance of integrating genetic information into lifestyle recommendations for effective obesity prevention.

Vitamin D metabolism genes and asthma susceptibility:

Rojo-Tolosa et al. identified a significant association between the Cdx2 genetic variant in vitamin D metabolism, and an increased risk of developing asthma. Their study underscores the role of vitamin D metabolism in respiratory health and suggests that genetic screening could guide supplementation or preventive strategies.

Genistein, miR-222, and muscle atrophy:

Gan et al. explored how the soy-derived phytoestrogen genistein combats dexamethasone-induced muscle wasting. Their findings show that genistein downregulates miR-222, which otherwise suppresses IGF1, a key muscle-growth factor. The study demonstrates a novel nutrient–miRNA interaction that could be harnessed to treat muscle atrophy.

TMEM18 gene predisposition to obesity and interaction with lifestyle factors:

Chermon & Birk explored the interaction between TMEM18 gene polymorphisms, alcohol intake, and physical activity in relation to obesity risk. Their findings reveal that regular physical activity and moderate alcohol consumption can attenuate the genetic predisposition to obesity in TMEM18 risk allele carriers, reinforcing the role of lifestyle in modulating genetic susceptibility.

Transcriptomic analysis of the anticancer effects of tocotrienols on chondrosarcoma cells:

Pang et al. studied the anticancer properties of vitamin E isoforms, assessing the transcriptomic impact of annatto-derived tocotrienols (δ- and γ-tocotrienol) on chondrosarcoma cells. The tocotrienols induced cell cycle arrest, apoptosis, and cytoplasmic vacuolation. Transcriptomic analysis revealed the upregulation of ER stress and autophagy-related pathways, and downregulation of oncogenic signaling such as Wnt and Hippo pathways. This research illustrates how natural compounds can modulate gene expression profiles, and supports their potential as nutrigenomic-based adjuvant therapies for cancer.

CYP2R1 and VDR polymorphisms and metabolic risk:

Pontes dos Santos et al. investigated CYP2R1 and VDR polymorphisms in non-diabetic overweight Brazilian adolescents. They identified significant associations between CYP2R1 variants and components of metabolic syndrome, particularly hyperglycemia, while no associations were found for VDR variants or serum 25(OH)D levels. *VDR* polymorphism was significantly associated with a risk of hypertension. These findings highlight a gene-specific contribution to metabolic risk, independent of vitamin D status.

Vitamin D metabolic pathway polymorphisms and the risk of non-small-cell lung cancer:

Pineda Lancheros et al. assessed the effect of 13 genetic polymorphisms in the vitamin D metabolic pathway on the risk of suffering from non-small-cell lung cancer (NSCLC). An observational case–control study found that carriers of the *VDR* BsmI rs1544410 and of the haplotype metabolic pathway of vitamin D were linked to lower NSCLC. These polymorphic markers could be of considerable value as a predictive disease biomarker. 

